# Alginate Nanoparticles Containing *Cuminum cyminum* and *Zataria multiflora* Essential Oils with Promising Anticancer and Antibacterial Effects

**DOI:** 10.1155/2024/5556838

**Published:** 2024-05-02

**Authors:** Mahmoud Osanloo, Razieh Ranjbar, Elham Zarenezhad

**Affiliations:** ^1^Department of Medical Nanotechnology, School of Advanced Technologies in Medicine, Fasa University of Medical Sciences, Fasa, Iran; ^2^Department of Medical Biotechnology, School of Advanced Technologies in Medicine, Fasa University of Medical Sciences, Fasa, Iran; ^3^Noncommunicable Disease Research Center, Fasa University of Medical Sciences, Fasa, Iran

## Abstract

Cancer and bacterial infections are major global health concerns driving the need for innovative medicines. This study investigated alginate nanoparticles loaded with essential oils (EOs) from *Cuminum cyminum* and *Zataria multiflora* as potential drug delivery systems. The nanoparticles were comprehensively characterized using techniques such as gas chromatography-mass spectrometry (GC-MS), dynamic light scattering (DLS), zetasizer, attenuated total reflectance-Fourier transform infrared spectroscopy (ATR-FTIR), and ultraviolet-visible spectroscopy (UV-Vis). Their biological properties against two human skin cancer cell lines (A-375 and A-431) and three bacteria (*Escherichia coli*, *Pseudomonas aeruginosa*, and *Staphylococcus aureus*) were also evaluated. Alginate nanoparticles containing *C. cyminum* and *Z. multiflora* EOs exhibited sizes of 160 ± 8 nm and 151 ± 10 nm, respectively. Their zeta potentials and encapsulation efficiencies were −18 ± 1 mV and 79 ± 4%, as well as −27 ± 2 mV and 86 ± 5%, respectively. The IC_50_ values against the tested cell lines and bacteria revealed superior efficacy for nanoparticles containing *Z. multiflora* EO. Considering the proper efficacy of the proposed nanoparticles, the straightforward preparation method and low cost suggest their potential for further in vivo studies.

## 1. Introduction

Skin cancer, a prevalent health concern, encompasses various types such as melanoma and nonmelanoma [1]. Researchers utilize human skin cancer cell lines A-375 (melanoma) and A-431 (epidermoid carcinoma) for anticancer studies due to their rapid growth [2–4]. Similarly, bacterial infections caused by *Escherichia coli*, *Pseudomonas aeruginosa*, and *Staphylococcus aureus* pose significant challenges. *E. coli*, commonly associated with UTIs and food-borne illness, can evolve into dangerous strains [5]. *P. aeruginosa* thrives in healthcare settings, causing critical infections, while *S. aureus*, a common skin colonizer, can become an antibiotic-resistant MRSA [6, 7].

The everescalating threat of antibiotic resistance and the seemingly never-ending battle against cancer have created a dire need for groundbreaking therapeutic strategies. In this urgent quest, exploring safe and effective herbal medicines offers a glimmer of hope [8, 9]. Plants such as *Zataria multiflora* and *Cuminum cyminum* have emerged as particularly exciting candidates due to their demonstrated anticancer and antimicrobial properties. However, a key challenge lies in harnessing their full therapeutic potential, as their essential oils (EOs) are susceptible to volatility and degradation [10, 11]. Alginate nanoparticles address the limitations of free EOs by offering a multitude of advantages. These nanoparticles can be engineered for controlled release, protecting EOs from degradation and improving their stability [12, 13]. In addition, alginate nanoparticles improve EO bioavailability by increasing water solubility, facilitating cellular uptake, and enhancing delivery through the EPR effect. This phenomenon, dependent on the nanoparticles' nanoscale size, concentrates the drug in the leaky vasculature of tumors, minimizing exposure to healthy tissues and reducing side effects [14, 15]. Furthermore, alginate nanoparticles are biocompatible, biodegradable, and versatile for various therapeutic applications. This combination of properties leads to improved therapeutic efficacy and bioavailability of EOs, potentially requiring lower doses for desired effects [16, 17].

Therefore, this study investigated alginate nanoparticles loaded with *C. cyminum* and *Z. multiflora* EOs as potential drug delivery systems. A comprehensive evaluation was applied to their anticancer activity against A-375 and A-431 cell lines, alongside their antibacterial activity against *E. coli*, *P. aeruginosa*, and *S. aureus*.

## 2. Materials and Methods

### 2.1. Materials

Commercially procured alginate sodium and calcium chloride were purchased from Sigma-Aldrich (USA). EOs of *C. cyminum* and *Z. multiflora* were purchased from Zardband Pharmaceuticals (Iran). Human cell lines of malignant melanoma (A-375, CRL-1619) and epidermoid carcinoma (A-431, CRL-1555) were obtained from the Pasteur Institute of Iran, along with several bacterial strains, i.e., *E. coli* (ATCC 25922), *P. aeruginosa* (ATCC 27853), and *S. aureus* (ATCC 25923).

### 2.2. Preparation of Alginate Nanoparticles Containing *C. cyminum* and *Z. multiflora* EOs

The ionic gelation method was used to prepare nanoparticles containing EOs [18]. In brief, a mixture of 0.25% w/v of each EO with 0.2% w/v of Tween 20 was stirred (2000 rpm, RT, 3 min). After that, the aqueous alginate solution (0.25% w/v) was gradually added and was stirred for 5 min. Finally, the aqueous solution of calcium chloride (0.04% w/v for *C. cyminum* EO and 0.05% w/v for *Z. multiflora*) was added dropwisely and stirred for 40 min for performing reactions of calcium ions with hydroxyl groups in alginates. The prepared nanoparticles containing *C. cyminum* and *Z. multiflora* EOs were named Alg-CC and Alg-ZM. In addition, alginate particles were prepared following the same procedure but without EO; this sample was named Alg-free.

### 2.3. Instrumental Analyses

#### 2.3.1. GC-MS

This analysis confirmed the chemical composition of the original *C. cyminum* and *Z. multiflora* EOs. An Agilent 6890 gas chromatograph equipped with a BPX5 column (3 mm × 0.52 mm ID × 52 *μ*m) and an Agilent 5973 mass spectrometer were used. The column temperature program started at 23°C (hold for 2 min) and then increased to 250°C at 10°C/min, followed by a further increase to 300°C at 12°C/min (hold for 5 min). Helium was used as the carrier gas (1.5 mL/min). The mass spectra were compared to the literature and library data for identification.

#### 2.3.2. DLS and Zeta Potential

The size distribution (hydrodynamic diameter) and zeta potential of Alg-CC and Alg-ZM were analyzed using a k-one (Nano, Ltd., Korea) DLS device. Zeta potential was further measured using a Horiba SZ-100 (Japan) zetasizer. Nanoparticles with a size below 200 nm and a SPAN less than 1, along with a positive or negative zeta potential value (indicating sufficient surface charge), were considered suitable.

#### 2.3.3. ATR-FTIR

The ATR-FTIR spectroscope (Bruker, Tensor II, Germany) investigated the chemical characteristics of sodium alginate, *C. cyminum* and *Z. multiflora* EOs, Alg-free, Alg-CC, and Alg-ZM at room temperature in the wavenumber range of 500–3500 cm^−1^. ATR-FTIR spectroscopy was utilized to confirm the loading of EOs in nanoparticles qualitatively.

#### 2.3.4. UV-Vis

The encapsulation efficiency of EOs within the nanoparticles was determined using UV-Vis spectroscopy. A standard calibration curve was established by measuring the absorbance of various concentrations (5–200 *μ*g/mL) of *C. cyminum* and *Z. multiflora* EOs in absolute ethanol at a common *λ*max (wavelength of maximum absorption). Linear regression analysis (*R*^2^ > 0.95) confirmed the accuracy of the calibration curve. Subsequently, Alg-CC and Alg-ZM nanoparticles were centrifuged, and the unencapsulated EO in the supernatant was quantified using the established calibration curve. Encapsulation efficiency was then calculated using the following equation: ((initial EO concentration) - (EO concentration in supernatant))/(initial EO concentration) × 100%.

### 2.4. Cytotoxic Effects of Nanoparticles

The MTT assay was used to assess the cytotoxicity of Alg-CC and Alg-ZM against A-375 and A-431 cells. Cells (10,000 cells/well) were seeded in 96-well plates and cultured for 24 hours to reach 80% confluency. The culture medium was then replaced with fresh medium containing various concentrations (39–1250 *μ*g/mL) of Alg-CC, Alg-ZM, or controls (PBS and Alg-free). Following another 24 hours of incubation, the cells were treated with MTT solution for 4 hours. The formed formazan crystals were then dissolved in DMSO, and the absorbance was measured at 570 nm using a plate reader. Cell viability was calculated by comparing the absorbance of treated cells to the control group, providing insights into the cytotoxic potential of these nanoparticles.

### 2.5. Antibacterial Effects of Nanoparticles

The antibacterial potential of Alg-CC and Alg-ZM against *E. coli*, *P. aeruginosa*, and *S. aureus* was evaluated using a microdilution assay. Standardized bacterial suspensions (0.5 McFarland) were incubated with serially diluted Alg-CC and Alg-ZM solutions (39–1250 *μ*g/mL) in 96-well plates. Control wells included PBS and Alg-free for comparison. Bacterial growth was assessed after 24 hours of incubation by measuring the optical density of each well at 630 nm. This assay provided insights into the effectiveness of these nanoparticles against various bacterial pathogens.

### 2.6. Statistical Analyses

All experiments were performed in triplicate, and data were presented as the mean ± standard deviation. Independent samples' *t*-tests analyzed the cytotoxicity and antibacterial effects at various concentrations. GraphPad Prism software facilitated data analysis and visualization, while CalcuSyn software (free version) determined the IC_50_ values for each sample.

## 3. Results

### 3.1. Identified Compounds

GC-MS analysis identified about 96% and 99% of the compounds in *C. cyminum* and *Z. multiflora* EOs, respectively. The identified compounds are listed in Table 1. Cumin aldehyde (42.2%), ortho-cymen (34.2%), and *β*-pinene (10.8%) were the three major constituents of *C. cyminum* EO. In comparison, carvacrol (38.7%), thymol (26.5%), and ortho-cymen (11.6%) were the most abundant compounds in *Z. multiflora* EO.

### 3.2. Prepared Nanoparticles

**Figure 1:**
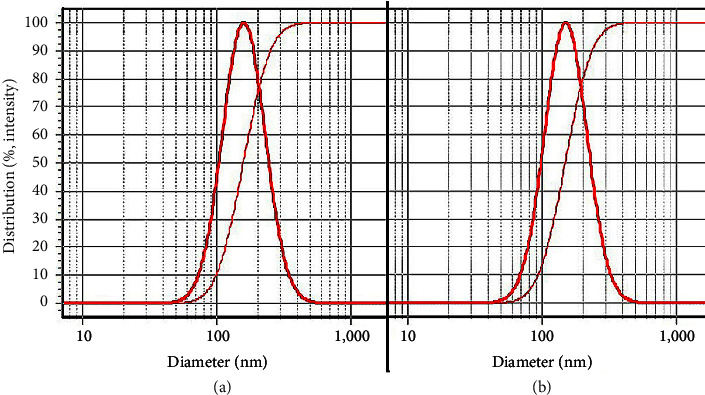
DLS profiles of (a) Alg-CC and (b) Alg-ZM.

**Figure 2:**
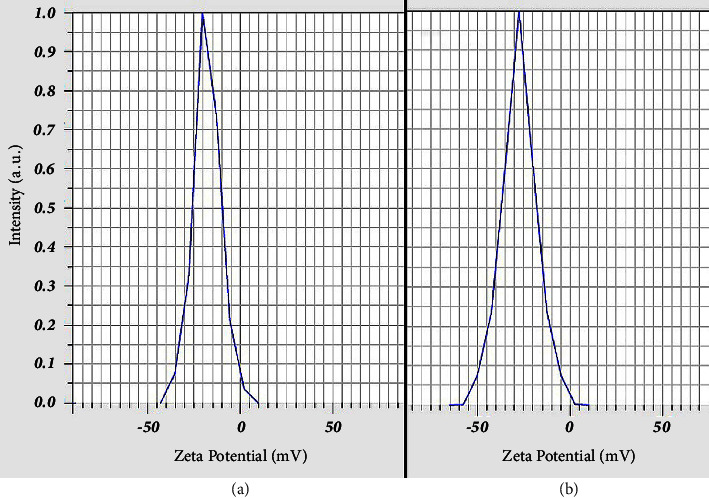
Zeta potential profiles of (a) Alg-CC and (b) Alg-ZM.

DLS analysis revealed that Alg-CC and Alg-ZM nanoparticles possessed hydrodynamic diameters of 160 ± 8 nm and 151 ± 10 nm, respectively, with narrow size distributions (SPAN values of 0.96 and 0.97), as shown in Figure 1. In addition, zeta potential measurements (Figure 2) indicated negative surface charges of −18 ± 1 mV and −27 ± 2 mV for Alg-CC and Alg-ZM, respectively.

### 3.3. Confirming Successful Loading of EOs in Nanoparticles

**Figure 3:**
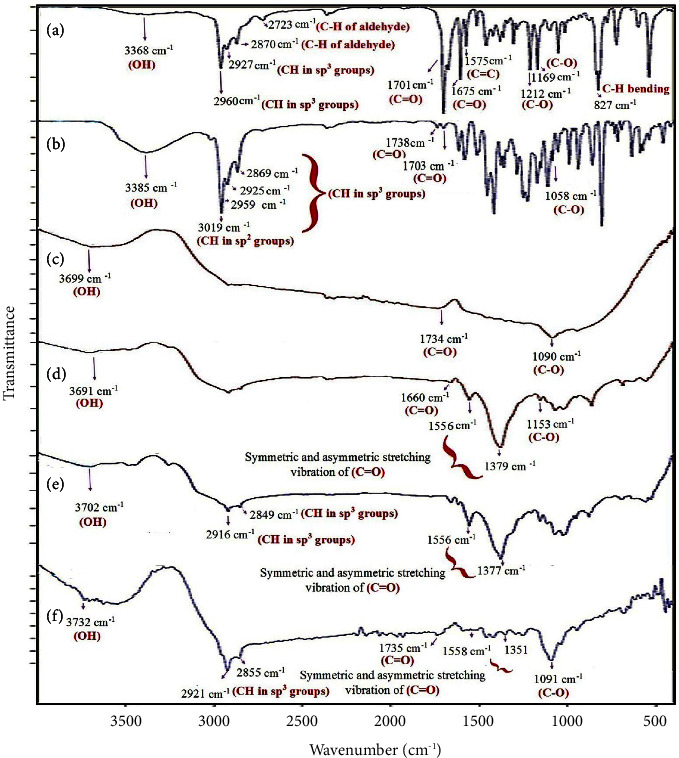
ATR-FTIR spectra of (a) *C. cyminum* EO, (b) *Z. multiflora* EO, (c) Alg-free, (d) Alg-free, (e) Alg-CC, and (f) Alg-ZM.

The ATR-FTIR spectra of *C. cyminum* EO (Figure 3(a)) showed a broad peak at 3368 cm^−1^, which is related to the stretching vibration of OH. The bands at 2960, 2927, and 2870 cm^−1^ showed the -CH stretching vibration of Sp^3^ in alkanes. The bands at 2870 and 2723 cm^−1^ indicate C-H of aldehyde. The characteristic bands at 1701 and 1675 cm^−1^ are assigned to EO's carbonyl stretching vibration in aldehyde and ketones. These strong peaks represented a high amount of aldehydes in the *L. C. cyminum* EO. The bands at 1575 and 1460 cm^−1^ showed the C=C vibration of an aromatic compound. The peaks 1169 and 1212 cm^−1^ are attributed to C-O stretching vibration. The band at 948 cm^−1^ is related to C-H bending absorption, and the strong peak at 827 cm^−1^ is assigned to benzene rings C-H vibration absorption. The peak at 687 cm^−1^ corresponds to the vibration absorption of alkenes.

ATR-FTIR spectra of *Z. multiflora* EO (Figure 3(b)) showing the broad band at 3200–3500 cm^−1^ can be attributed to stretching vibration of the hydroxyl group due to hydrogen bonding in phenolic and alcoholic compounds in EO, the band at 3019 cm^−1^ can be related to stretching vibration of CH in sp^2^ groups, the bands at 2959, 2925, and 2869 cm^−1^ can be corresponded to stretching vibration of CH in sp^3^ groups, and the bands at 1738 and 1703 cm^−1^ can be attributed to stretching vibration carbonyl groups. The spectra at 1088 and 1058 cm^−1^ displayed the stretching vibration of the C-O groups.

From Figure 3(c), the ATR-FTIR spectra of Alg-free (Alg-CC without *L. C. cyminum* EO) displayed the broad band between 3200 and 3699 cm^−1^ corresponding to OH stretching vibration due to hydrogen bonding in Tween, water, and alginate. The band at 1734 cm^−1^ can be attributed to the carbonyl group in Tween. The band at 1090 cm^−1^ can be related to C-O stretching vibration.

From Figure 3(d), the ATIR spectra of Alg-free (Alg-ZM without *Z. multiflora* EO) showed that the broadband between 3200 and 3691 cm^−1^ can be related to the stretching vibration of hydroxyl groups in Tween, water, and alginate. The band at 1660 cm^−1^ can be related to the stretching vibration of the carbonyl group in Tween 20. The bands at 1556 and 1379 cm^−1^ are related to the symmetric and asymmetric stretching vibration of carbonyl groups due to sodium alginate. The band at 1153 and 1069 cm^−1^ can be attributed to the stretching vibration of C-O groups.

The ATR-FTIR spectra of Alg-CC (Figure 3(e)) showed a peak at about 3254–3702 cm^−1^, which is assigned to OH. The peaks at 2916 and 2849 cm^−1^ corresponded to C-H stretching vibration. The bands at 1656 and 1617 cm^−1^ can be related to the presence of EO and Tween 20. The 1556 and 1377 cm^−1^ peaks can be attributed to carbonyl groups' symmetric and asymmetric stretching vibration.

The ATR-FTIR spectra of Alg-ZM (Figure 3(f)) showed a peak at about 3330–3732 cm^−1^, which is assigned to OH. The peaks at 2921 and 2855 cm^−1^ corresponded to C-H stretching vibration. The band at 1735 cm^−1^ can be related to C=O, the presence of EO, and Tween 20. The 1558 and 1351 cm^−1^ peaks can be attributed to carbonyl groups' symmetric and asymmetric stretching vibration. The stretching and characteristic band at 1091 cm^−1^ in nanoparticles containing EOs corresponds to the reaction between carboxyl and Ca ion (CO-Ca-CO group structure), increasing C-O vibration. This peak demonstrated that ionic crosslinking and physical crosslinking between the hydroxyl groups of alginate, Tween 20, and EO also consume a small amount of hydroxyl groups.

### 3.4. Encapsulation Efficacy of EOs in Nanoparticles

**Figure 4:**
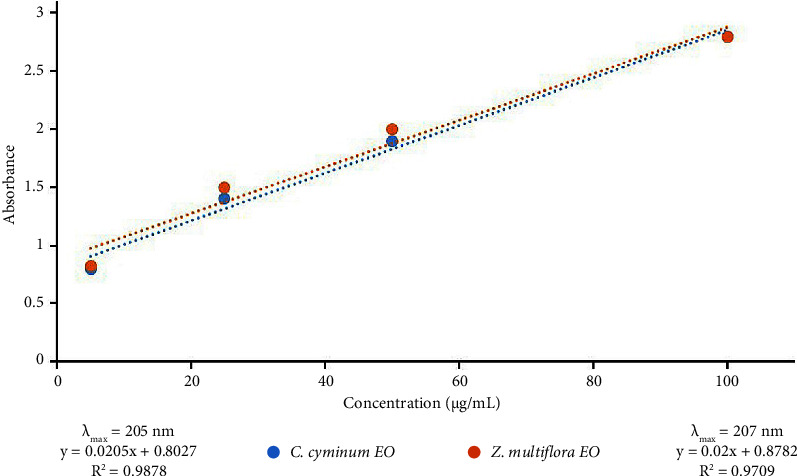
Calibration curves of *C. cyminum* and *Z. multiflora* EOs and their regression equations.

The established calibration curves for *C. cyminum* and *Z. multiflora* EOs (Figure 4) enabled the quantification of encapsulated EOs within the nanoparticles. Encouragingly, Alg-CC and Alg-ZM displayed high encapsulation efficiencies of 79 ± 4% and 86 ± 5%, respectively.

### 3.5. Cytotoxic Effects of Nanoparticles

**Figure 5:**
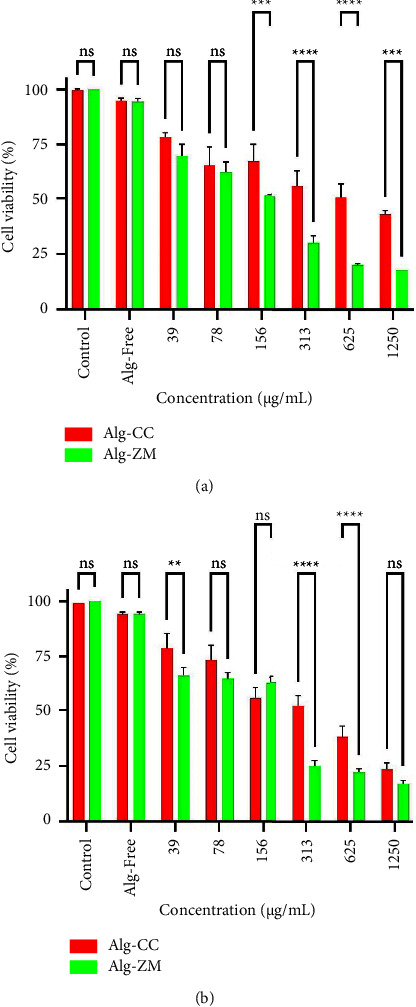
Cytotoxic effects of Alg-CC and Alg-ZM against (a) A-375 and (b) A-431 cells. ns: not significant, ^*∗∗*^*P* < 0.01, ^*∗∗∗*^*P* < 0.001, and ^*∗∗∗∗*^*P* < 0.0001.

As shown in Figure 5, Alg-free treatment resulted in minimal cytotoxicity (viability >90%) towards both A-375 and A-431 cells, indicating negligible effects of the carrier material. Notably, Alg-ZM exhibited significantly higher cytotoxicity than Alg-CC against both cell lines. The IC_50_ values of Alg-ZM for A-375 and A-431 cells were 132 (104–167) *μ*g/mL and 158 (102–247) *μ*g/mL, respectively (Table 2). In contrast, Alg-CC displayed lower cytotoxicity, with IC_50_ values of 664 (439–1005) *μ*g/mL and 321 (247–416) *μ*g/mL for A-375 and A-431 cells, respectively.

### 3.6. Antibacterial Effects of Nanoparticles

**Figure 6:**
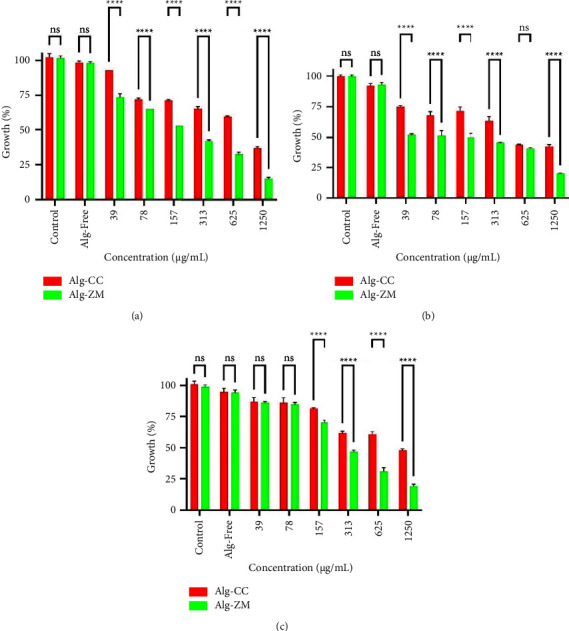
Antibacterial effects of Alg-CC and Alg-ZM against (a) *E. coli*, (b) *P. aeruginosa*, and (c) *S. aureus*. ns: not significant and ^*∗∗∗∗*^*P* < 0.0001.

As illustrated in Figure 6, Alg-free treatment exhibited negligible antibacterial activity against all three bacterial strains (*E. coli*, *P. aeruginosa*, and *S. aureus*), indicating the carrier material's lack of inherent antimicrobial properties. Encouragingly, Alg-ZM demonstrated significantly greater antibacterial efficacy than Alg-CC against all tested bacteria. The IC_50_ values of Alg-ZM for *E. coli*, *P. aeruginosa*, and *S. aureus* were 178 (145–219) *μ*g/mL, 95 (38–238) *μ*g/mL, and 307 (252–375) *μ*g/mL, respectively (Table 2). In contrast, Alg-CC displayed lower antibacterial activity, with IC_50_ values of 690 (344–1381) *μ*g/mL, 633 (347–1153) *μ*g/mL, and 1098 (637–1794) *μ*g/mL for *E. coli*, *P. aeruginosa*, and *S. aureus*, respectively.

## 4. Discussion

Alginate nanoparticles have been explored as a delivery system for various EOs, including *Z. multiflora* EO, for its antioxidant activity [19]. Besides, other nanocarriers, including nanoliposomes and solid lipid nanoparticles, were proposed for their larvicidal activity [20, 21] and mosquito-repellent properties [22]. Similarly, *C. cyminum* EO-loaded nanoformulations have been investigated for their antioxidant, anticancer, and antibacterial properties [23]. Interestingly, a previous study also employed alginate nanoparticles containing a blend of *C. cyminum* and *Z. multiflora* EOs as a shrimp coating, demonstrating its potential for combined effects [24]. This suggests a precedent for the safety of Alg-CC and Alg-ZM based on the similarity of their components.

Our study demonstrates the potent efficacy of Alg-ZM compared to Alg-CC against cancer cells and bacteria. The obtained IC_50_ value (132 *μ*g/mL) for Alg-ZM against A-375 cells is comparable or superior to previously reported values for alginate nanoparticles containing other EOs. For instance, IC_50_ values of alginate nanoparticles containing clove EO and eugenol on A-375 were reported as 358 and 758 *μ*g/mL [25]. The effects of alginate nanoparticles containing *Lavandula angustifolia* EO on A-375 were reported: IC_50_ = 405 *μ*g/mL [18]. Moreover, the IC_50_ value of nanoemulsion containing *Origanum majorana* EO was reported as 139 *μ*g/mL [26]. Similarly, Alg-ZM exhibited superior antibacterial activity against *P. aeruginosa* (IC_50_ = 95 *μ*g/mL) compared to previously reported nanoemulsions and nanogels containing *Eucalyptus globulus* EO with IC_50_ value of >5000 *μ*g/mL [25].

While the exact mechanism for Alg-CC and Alg-ZM's activity remains elusive, studies suggest potential pathways based on the individual EOs and their major components. *Z. multiflora* EO has been shown to induce apoptosis in cancer cells via the mitochondrial pathway and generation of reactive oxygen species (ROS) [27]. *C. cyminum* EO might inhibit bacterial biofilm formation and virulence factors [28]. More generally, EOs' complex chemical composition can contribute to various therapeutic activities, including disrupting bacterial membranes, inhibiting enzymes, and interfering with protein synthesis [29–31]. EOs might also exhibit antioxidant properties and antibiotic synergistic effects [32, 33].

Given the presence of carvacrol and thymol as the major components in *Z. multiflora* EO, their mechanisms of action are particularly relevant. Both carvacrol and thymol have demonstrated anticancer and antibacterial properties. They can induce apoptosis and cell cycle arrest and inhibit proliferation in cancer cells [34–36]. Their antibacterial effects involve disrupting bacterial membranes, inhibiting efflux pumps, and interfering with protein synthesis [37–39]. Carvacrol and thymol can act synergistically with conventional antibiotics [40, 41]. However, no information was found on the anticancer and antibacterial effects of cuminic aldehyde, a major component of *C. cyminum* EO.

## 5. Conclusion

This study explored alginate nanoparticles (Alg-CC and Alg-ZM) encapsulating *C. cyminum* and *Z. multiflora* EOs as a cytotoxic and antibacterial agent delivery system. The results were promising, with Alg-ZM demonstrating significantly higher efficacy against A-375 cancer cells and *P. aeruginosa* relative to other reported nanoformulations. However, this study demonstrates promising in vitro efficacy of alginate nanoparticles loaded with two EOs against cancer cells and bacteria; limitations include the lack of in vivo testing and focus on a narrow range of cell lines and bacteria. Future research should investigate the mechanism of action and conduct in vivo studies with broader cell lines and bacterial strain selection to solidify the potential of these nanoparticles as a drug delivery system for cancer and bacterial infections.

## Figures and Tables

**Table 1:** Identified compounds in the EOs using GC-MS analysis.

Retention time (min)	Compounds	*C. cyminum* %	*Z. multiflora* %	Kovats index	Type
11.2	*α*-Thujene	—	0.2	930	MH
11.6	*α*-Pinene	0.8	3.2	939	MH
12.5	Camphene	—	0.1	954	MH
14.0	*β*-Pinene	10.8	0.3	979	MH
14.5	*α*-Myrcene	—	1.2	991	MH
16.0	*α*-Terpinene	—	0.8	1017	MH
16.5	Ortho-cymen	34.2	11.6	1029	MH
16.7	Limonene	0.4	0.6	1029	MH
16.8	Eucalyptol	—	2.5	1031	MO
18.2	*γ*-Terpinene	2.3	2.4	1060	MH
20.4	Linalool	0.9	—	1097	MO
24.3	Borneol	—	0.1	1169	MO
24.6	Terpinene-4-ol	—	0.8	1177	MO
25.4	*α*-Terpineol	—	0.7	1188	MO
26.9	Thymol methyl ether	—	0.5	1235	MO
27.3	Carvacrol methyl ether	—	1.0	1244	MO
27.9	Cumin aldehyde	42.2	—	1241	MO
28.1	Carvotanacetone	0.4	—	1247	MO
29.5	Bornyl acetate	—	0.2	1285	MO
30.0	2.Caren-10-ol	2.4	—	1309	MO
30.0	Thymol	—	26.5	1290	MO
30.4	Carvacrol	0.5	38.7	1299	MO
32.2	Thymol acetate	—	1.0	1352	MO
33.1	Carvacrol acetate	—	1.7	1372	MO
35.3	Caryophyllene	—	1.4	1419	SH
36.2	Aromadendrene	—	0.9	1441	SH
37.7	Farnesene	1.1	—	1505	SH
38.4	Viridiflorenne	—	0.5	1496	SH
42.0	Spathulenol	—	0.6	1578	SO
42.2	Caryophyllene oxide	—	1.2	1583	SO
	Total identified	95.8	98.5		

MH: monoterpene hydrocarbons; MO: oxygenated monoterpenes; SH: sesquiterpene hydrocarbons; and SO: oxygenated sesquiterpenes SO.

**Table 2:** Obtained IC_50_ values (*μ*g/mL) of Alg-CC and Alg-ZM.

Samples	Factors	A-375	A-431	*E. coli*	*P. aeruginosa*	*S. aureus*
Alg-CC	IC_50_	664	321	690	633	1098
	LCL-UCL	439–1005	247–416	344–1381	347–1153	673–1794

Alg-ZM	IC_50_	132	158	178	95	307
	LCL-UCL	104–167	102–244	145–219	38–238	252–375

## Data Availability

The datasets used and/or analyzed during the current study are available from the corresponding author upon reasonable request.

## References

[1] Fraiwan M., Faouri E. (2022). On the automatic detection and classification of skin cancer using deep transfer learning. *Sensors*.

[2] Leupold D., Pfeifer L., Hofmann M., Forschner A., Wessler G., Haenssle H. (2021). From melanocytes to melanoma cells: characterization of the malignant transformation by four distinctly different melanin fluorescence spectra (review). *International Journal of Molecular Sciences*.

[3] Jeon S., Jeon M., Choi S. (2023). Hypoxia in skin cancer: molecular basis and clinical implications. *International Journal of Molecular Sciences*.

[4] Cheng S. L., Huang-Liu R., Sheu J. N., Chen S. T., Sinchaikul S., Tsay G. J. (2007). Toxicogenomics of A375 human malignant melanoma cells. *Pharmacogenomics*.

[5] Kaper J. B., Nataro J. P., Mobley H. L. T. (2004). Pathogenic *Escherichia coli*. *Nature Reviews Microbiology*.

[6] Pachori P., Gothalwal R., Gandhi P. (2019). Emergence of antibiotic resistance *Pseudomonas aeruginosa* in intensive care unit; a critical review. *Genes & Diseases*.

[7] Pal R., Tsering D., Kar S. (2011). Methicillin-resistant *Staphylococcus aureus*: prevalence and current susceptibility pattern in Sikkim. *Journal of Global Infectious Diseases*.

[8] Li T., Wang Z., Guo J. (2023). Bacterial resistance to antibacterial agents: mechanisms, control strategies, and implications for global health. *The Science of the Total Environment*.

[9] Sharma A. N., Dewangan H. K., Upadhyay P. K. (2024). Comprehensive review on herbal medicine: emphasis on current therapy and role of phytoconstituents for cancer treatment. *Chemistry and Biodiversity*.

[10] Ghorani V., Beigoli S., Khazdair M. R., Boskabady M. H. (2022). The effect of Zataria multiflora on respiratory allergic and immunologic disorders, experimental and clinical evidence: a comprehensive review. *Phytotherapy Research*.

[11] Mnif S., Aifa S. (2015). Cumin (Cuminum cyminum L.) from traditional uses to potential biomedical applications. *Chemistry and Biodiversity*.

[12] Pontillo A. R. N., Detsi A. (2019). Nanoparticles for ocular drug delivery: modified and non-modified chitosan as a promising biocompatible carrier. *Nanomedicine*.

[13] Gholamali I., Yadollahi M. (2021). Bio-nanocomposite polymer hydrogels containing nanoparticles for drug delivery: a review. *Regen Eng Transl Med*.

[14] Severino P., da Silva C. F., Andrade L. N., de Lima Oliveira D., Campos J., Souto E. B. (2019). Alginate nanoparticles for drug delivery and targeting. *Current Pharmaceutical Design*.

[15] He L., Shang Z., Liu H., Yuan Z. X. (2020). Alginate-based platforms for cancer-targeted drug delivery. *BioMed Research International*.

[16] Rastogi P., Kandasubramanian B. (2019). Review of alginate-based hydrogel bioprinting for application in tissue engineering. *Biofabrication*.

[17] Zhang H., Cheng J., Ao Q. (2021). Preparation of alginate-based biomaterials and their applications in biomedicine. *Marine Drugs*.

[18] Valizadeh A., Hosseinzadeh M., Heiran R., Hatami S., Hosseinipour A., Osanloo M. (2024). Alginate nanoparticles containing Lavandula angustifolia essential oil as a potential potent, biocompatible and low-cost antitumor agent. *Polymer Bulletin*.

[19] Hashemi M., Shakerardekani A., Mirzaalian Dastjerdi A., Mirdehghan S. (2021). Effect of sodium alginate in combination with *Zataria multiflora* boiss. On phenolic compounds, antioxidant activity, and browning enzymes of fresh in-hull pistachio (*Pistacia vera* L.). *Journal of Food Quality*.

[20] Sanei-Dehkordi A., Heiran R., Moemenbellah-Fard M. D., Sayah S., Osanloo M. (2022). Nanoliposomes containing carvacrol and carvacrol-rich essential oils as effective mosquitoes larvicides. *BioNanoScience*.

[21] Sanei-Dehkordi A., Agholi M., Shafiei M., Osanloo M. (2022). Promising larvicidal efficacy of solid lipid nanoparticles containing mentha longifolia L., mentha pulegium L., and Zataria multiflora boiss. Essential oils against the main malaria vector, *Anopheles stephensi* liston. *Acta Parasitologica*.

[22] Moemenbellah-Fard M. D., Firoozian S., Shahriari-Namadi M., Zarenezhad E., Roozitalab G., Osanloo M. (2022). A natural nanogel with higher efficacy than a standard repellent against the primary malaria mosquito vector, *Anopheles stephensi* Liston. *Chemical Papers*.

[23] Ranjbar R., Zarenezhad E., Abdollahi A. (2023). Nanoemulsion and nanogel containing Cuminum cyminum L essential oil: antioxidant, anticancer, antibacterial, and antilarval properties. *Journal of Tropical Medicine*.

[24] Osanloo M., Eskandari Z., Zarenezhad E., Qasemi H., Nematollahi A. (2023). Studying the microbial, chemical, and sensory characteristics of shrimp coated with alginate sodium nanoparticles containing Zataria multiflora and Cuminum cyminum essential oils. *Food Science and Nutrition*.

[25] Yarian F., Yousefpoor Y., Hatami S. (2023). Comparison effects of alginate nanoparticles containing syzygium aromaticum essential oil and eugenol on apoptotic regulator genes and viability of A-375 and MCF-7 cancer cell lines. *BioNanoScience*.

[26] Rasti F., Ahmadi E., Safari M. (2023). Anticancer, antioxidant, and antibacterial effects of nanoemulsion of Origanum majorana essential oil. *Iranian Journal of Microbiology*.

[27] Ahani N., Sangtarash M. H., Alipour Eskandani M., Houshmand M. (2020). Zataria multiflora boiss. Essential oil induce apoptosis in two human colon cancer cell lines (HCT116 & SW48). *Iranian Journal of Public Health*.

[28] Ghannay S., Aouadi K., Kadri A., Snoussi M. (2022). In vitro and in silico screening of anti-Vibrio spp., antibiofilm, antioxidant and anti-quorum sensing activities of Cuminum cyminum L. Volatile oil. *Plants*.

[29] Amala Dev A. R., Sonia Mol J. (2023). Citrus essential oils: a rational view on its chemical profiles, mode of action of anticancer effects/antiproliferative activity on various human cancer cell lines. *Cell Biochemistry and Biophysics*.

[30] He Y., Sang S., Tang H., Ou C. (2022). In vitro mechanism of antibacterial activity of Eucalyptus essential oil against specific spoilage organisms in aquatic products. *Journal of Food Processing and Preservation*.

[31] Truong S., Mudgil P. (2023). The antibacterial effectiveness of lavender essential oil against methicillin-resistant *Staphylococcus aureus*: a systematic review. *Frontiers in Pharmacology*.

[32] Chen X., Shang S., Yan F. (2023). Antioxidant activities of essential oils and their major components in scavenging free radicals, inhibiting lipid oxidation and reducing cellular oxidative stress. *Molecules*.

[33] Tosun M. N., Taylan G., Demirel Zorba N. N. (2022). Antibacterial and antibiofilm activities of some plant essential oils and synergistic effects of cinnamon essential oil with vancomycin against Clostridioides difficile: in vitro study. *Letters in Applied Microbiology*.

[34] Dikić J., Lukić I., Pajnik J., Pavlović J., Hrenović J., Rajić N. (2021). Antibacterial activity of thymol/carvacrol and clinoptilolite composites prepared by supercritical solvent impregnation. *Journal of Porous Materials*.

[35] Sampaio L. A., Pina L. T. S., Serafini M. R., Tavares D. D. S., Guimarães A. G. (2021). Antitumor effects of carvacrol and thymol: a systematic review. *Frontiers in Pharmacology*.

[36] Qoorchi Moheb Seraj F., Heravi-Faz N., Soltani A. (2022). Thymol has anticancer effects in U-87 human malignant glioblastoma cells. *Molecular Biology Reports*.

[37] Islam M. T., Khalipha A. B. R., Bagchi R. (2019). Anticancer activity of thymol: a literature-based review and docking study with Emphasis on its anticancer mechanisms. *IUBMB Life*.

[38] Kowalczyk A., Przychodna M., Sopata S., Bodalska A., Fecka I. (2020). Thymol and thyme essential oil-new insights into selected therapeutic applications. *Molecules*.

[39] Marchese A., Orhan I. E., Daglia M. (2016). Antibacterial and antifungal activities of thymol: a brief review of the literature. *Food Chemistry*.

[40] Kachur K., Suntres Z. (2020). The antibacterial properties of phenolic isomers, carvacrol and thymol. *Critical Reviews in Food Science and Nutrition*.

[41] Raei P., Pourlak T., Memar M. Y. (2017). Thymol and carvacrol strongly inhibit biofilm formation and growth of carbapenemase-producing Gram negative bacilli. *Cellular & Molecular Biology*.

